# Islet neogenesis associated protein (INGAP) protects pancreatic β cells from IL-1β and IFNγ-induced apoptosis

**DOI:** 10.1038/s41420-021-00441-z

**Published:** 2021-03-17

**Authors:** Eni Nano, Maria Petropavlovskaia, Lawrence Rosenberg

**Affiliations:** Lady Davis Institute for Medical Research, SMBD-Jewish General Hospital, Department of Surgery, Faculty of Medicine, McGill University, 3755, Cote Ste-Catherine Rd, Montreal, QC H3T 1E2 Canada

**Keywords:** Mechanisms of disease, Inflammation

## Abstract

The goal of this study was to determine whether recombinant Islet NeoGenesis Associated Protein (rINGAP) and its active core, a pentadecapeptide INGAP^104–118^ (Ingap-p), protect β cells against cytokine-induced death. INGAP has been shown to induce islet neogenesis in diabetic animals, to stimulate β-cell proliferation and differentiation, and to improve islet survival and function. Importantly, Ingap-p has shown promising results in clinical trials for diabetes (phase I/II). However, the full potential of INGAP and its mechanisms of action remain poorly understood. Using rat insulinoma cells RINm5F and INS-1 treated with interleukin-1β (IL-1β) and interferon‐gamma (IFN‐γ), we demonstrate here that both rINGAP and Ingap-p inhibit apoptosis, Caspase-3 activation, inducible nitric oxide synthase (iNOS) expression and nitric oxide (NO) production, and explore the related signaling pathways. As expected, IL-1β induced nuclear factor kappa B (NF-κB), p38, and JNK signaling, whereas interferon‐gamma (IFN‐γ) activated the JAK2/STAT1 pathway and potentiated the IL-1β effects. Both rINGAP and Ingap-p decreased phosphorylation of IKKα/β, IkBα, and p65, although p65 nuclear translocation was not inhibited. rINGAP, used for further analysis, also inhibited STAT3, p38, and JNK activation. Interestingly, all inhibitory effects of rINGAP were observed for the cytokine cocktail, not IL-1β alone, and were roughly equal to reversing the potentiating effects of INFγ. Furthermore, rINGAP had no effect on IL-1β/NF-κB-induced gene expression (e.g., Ccl2, Sod2) but downregulated several IFNγ-stimulated (Irf1, Socs1, Socs3) or IFNγ-potentiated (Nos2) genes. This, however, was observed again only for the cytokine cocktail, not IFNγ alone, and rINGAP did not inhibit the IFNγ-induced JAK2/STAT1 activation. Together, these intriguing results suggest that INGAP does not target either IL-1β or IFNγ individually but rather inhibits the signaling crosstalk between the two, the exact mechanism of which remains to be investigated. In summary, our study characterizes the anti-inflammatory effects of INGAP, both protein and peptide, and suggests a new therapeutic utility for INGAP in the treatment of diabetes.

## Introduction

Diabetes mellitus is a metabolic disorder characterized by β-cell deficiency and the resulting hyperglycemia. In Type 1 diabetes (T1DM), β cells are destroyed by autoimmunity^1^, whereas in Type 2 diabetes (T2DM), β-cell failure and death come from progressive insulin resistance, metabolic and oxidative stress, and persistent hyperglycemia^2^.

Both T1DM and T2DM are characterized by islet inflammation, hyperglycemia, and β-cell apoptosis^3,4^. Studies in rodents and humans pinpoint pro-inflammatory cytokines and chemokines as key mediators of T1DM pathophysiology and as contributing factors to insulin resistance and β-cell dysfunction in T2DM^5,6^. Pro-inflammatory cytokines, interleukin-1β (IL-1β), tumor necrosis factor alpha (TNFα), and interferon‐gamma (IFN‐γ), have been reported to trigger destructive signaling pathways within β cells, leading to elevation of reactive oxygen species, β-cell dysfunction, and eventually to apoptosis^5–7^. Inhibition of these signaling pathways is therefore an important therapeutic objective aimed at preventing the progression of diabetes and preserving β-cell mass. Protection of β cells is also essential for regenerative therapies to enable survival and function of transplanted islets or stem cell-generated β cells. In this context, identification of factors with anti-inflammatory, anti-apoptotic and regenerative effects, is of interest.

The goal of this study was to investigate whether INGAP (Islet NeoGenesis Associated Protein), the first known regenerating agent with islet neogenic properties, protects β cells against pro-inflammatory cytokines. INGAP, a 16.8 kDa Reg3δ protein, was discovered in a partially obstructed hamster pancreas as a factor inducing duct-associated islet neogenesis^8–12^. Several studies have indicated that the islet neogenic activity of INGAP protein is mediated by a pentadecapeptide (Ingap-p, aa104–118)^11,13–16^, which achieves the same effect, albeit at higher concentrations^11,16–18^. Importantly, Ingap-p has been proven safe to use in humans and has reached Phase 2 clinical trials. Daily injections of Ingap-p for 90 days improved glucose homeostasis and reduced A1C levels in T2DM patients, and significantly increased C-peptide in T1DM patients^19^.

Ingap-p has been used in numerous studies that corroborated its duct-to-β-cell neogenic activity^20,21^ and demonstrated its pleiotropic nature including stimulation of insulin secretion, islet survival and function ^22–29^, and angiogenesis ^30^. In addition to increasing functionality and survival of isolated rat islets^29,31,32^, Ingap-p has been shown to increase viability of islet allografts post-transplantation^33^.

The full-length INGAP protein expressed in the acinar tissue rendered mice resistant to streptozotocin (STZ)-induced diabetes^34^. The protective effects of another INGAP transgene expressed in β cells correlated with downregulation of NADPH oxidase NOX1, one of the main mediators of the STZ-induced oxidative damage and DNA fragmentation in islets, thus suggesting that INGAP possesses anti-oxidative properties^35^.

The reported protective effects of INGAP in isolated islets are particularly interesting, suggesting its potential utility for islet transplantation but the mechanisms underlying these effects are unknown. The isolation process causes enzymatic, ischemic, and mechanical stresses that negatively impact islet survival^36,37^, induce cytokines and chemokines in the islets and activate nuclear factor kappa B (NF-κB), resulting in a strong inflammatory response^38,39^.

The anti-inflammatory effects of INGAP have not been investigated. We hypothesized that INGAP may offset the cytotoxicity of pro-inflammatory cytokines in β cells and tested our hypothesis using two β-cell lines RINm5F and INS-1 treated with IL-1β and IFNγ, commonly used in the models of cytokine-induced β-cell death^40^. IL-1β exerts its effects predominantly through the NF-κB pathway^41^, whereas IFNγ acts mostly through the JAK/STAT1 pathway^42^ but may also activate STAT3^43,44^ and was shown to potentiate the effects of other cytokines^45–47^. NF-κB activation by IL-1β and IFNγ leads to stimulation of inducible nitric oxide synthase (iNOS) and production of nitric oxide (NO) directly implicated in β-cell apoptosis^46^. To characterize the anti-cytokine effects of INGAP and to investigate the underlying signaling events we used, as previously^17,18^, both INGAP-peptide and the full-length recombinant protein (rINGAP).

## Materials and methods

### Cell culture

RINm5F cells (ATCC) were cultured at 37°C in a humidified atmosphere of 5% CO_2_ in Roswell Park Memorial Institute (RPMI)-1640 medium (Gibco^TM^ Life Technologies, Burlington ON, Canada) containing 11 mM glucose, 10% fetal bovine serum (FBS), 10 mM HEPES (4-(2-hydroxyethyl)-1-piperazineethanesulfonic acid), 1 mM sodium pyruvate (Wisent, St-Bruno, QC, Canada) and 1x antibiotics/antimycotics (Gibco^TM^).

INS-1 cells (AddexBio, San Diego, CA, USA) were cultured in the same medium, supplemented with 50 μM β-mercaptoethanol (Sigma-Aldrich, Oakville, ON, Canada).

Cells passaged every 4–5 days, were plated in six-well (500,000 cells/well), 24-well (200,000 cells/well), or 96-well (40,000 cells/well) TC dishes for 48 h, and serum-starved for 24 h prior to the treatment.

### In vitro model of cytokine-induced β-cell death

To optimize the conditions for cytokine-induced death, RINm5F and INS-1 cells were treated with either 100 pg/mL IL-1β, 1 ng/mL IFNγ (R&D Systems, Minneapolis, MN, USA), or a cocktail of both and screened for (1) expression of Nos2 by real-time quantitative RT-PCR (qRT-PCR), (2) production of NO in the culture media by Griess assay, and (3) viability by 3-(4,5-dimethythiazol-2-yl)−2,5-diphenyl tetrazolium bromide (MTT) assay. All experiments were repeated at least three times.

As summarized in Supplementary Fig. 1, Nos2 expression in RINm5F cells (3 h and 6 h) and NO levels in both cell lines (24 h and 48 h) were slightly induced by IL-1β and significantly potentiated by IFNγ, which alone was ineffective (A–C*)*. Vice versa, IL-1β did not reduce viability, which was affected mostly by IFNγ (E). NO levels were significantly increased at 10× and 100× cytokine concentrations, whereas viability varied much less (D, F, G). We concluded that a cocktail of 100 pg/mL IL-1β+1 ng/mL IFNγ was sufficient to induce cytotoxicity in both cell lines.

### rINGAP and Ingap-p

Recombinant INGAP, MW 17.6 kDa, was produced in-house, as previously described^16^, and high performance liquid chromatography (HPLC)-purified (Sheldon Biotechnology Centre, McGill University, Montreal, QC, Canada). Ingap-p, MW 1501.6 Da, was synthesized and HPLC-purified (Sheldon Biotechnology Centre or CanPeptide (Pointe-Claire, QC, Canada)). As previously^17^, 1 nM rINGAP and 1.67 μM Ingap-p were used in most experiments.

### Assessment of viability and measurement of NO production

Viability (cellular metabolic activity) was measured by MTT assay. Following treatment in 96-well plates, 20 μL/well of 5 mg/mL MTT (Sigma-Aldrich) was added for 4 h at 37 °C, 5% CO_2_. Formazan pellets were solubilized overnight at 37 °C with 0.01 M HCl+10% SDS (100 μL/well). Absorbance was measured at 570 nm using microplate spectrophotometer (Bio-Rad Laboratories, Mississauga, ON, Canada). All samples were tested in triplicates and each experiment was repeated 4–5 times. NO production was measured in 50 μL of cell culture media (duplicate samples) using the Griess assay kit (Promega, Madison, WI, USA), according to the manufacturer’s instructions. Experiments were repeated three times.

### Assessment of apoptosis by Annexin-V/PI FACS analysis

Following treatment, cells were trypsinized, washed with 1× phosphate-buffered saline (PBS) and stained using the FITC Annexin-V Apoptosis Detection Kit with PI (BioLegend, San Diego, CA, USA), following the manufacturer’s instructions. Stained cells and controls (unstained cells, FITC Annexin-V only, and PI only) were then processed by fluorescence-activated cell sorting (FACS) (BD LSR Fortessa, LDI Flow Cytometry Services) and analyzed by FlowJo software. Each experiment was repeated three times.

### Real-time quantitative RT-PCR

Cells were lysed in RLT buffer for RNA extraction with RNeasy or RNeasy Plus Kits in QIAcube (Qiagen, Toronto, ON, Canada). One microgram of RNA was reverse-transcribed using the Omniscript kit (Qiagen). Quantitative PCR was performed using custom-made primers (Supplementary Table 1), iQ^TM^ SYBR^®^ Green master mix (Bio-Rad) in CFX96^TM^ Real-Time System (Bio-Rad). Normalized gene expression was calculated using the ΔΔC_T_ method and Gapdh or multiple reference genes (Gapdh, α-tubulin, and β-actin) by the CFX Manager^TM^ software (Bio-Rad). All experiments were repeated at least three to five times.

### Immunostaining

Cells (100,000/well) plated in eight-chambered glass slides coated with poly-d-lysine (Sigma-Aldrich) were fixed in 4% paraformaldehyde (10 min, room temperature). Apoptosis was assessed by the terminal deoxynucleotidyl transferase-mediated dUTP nick-end labeling (TUNEL) method using APO-BrdU^TM^ TUNEL Assay Kit (Invitrogen^TM^, Life Technologies). Staining for cleaved caspase-3 with primary antibody (rabbit, 1:100, Cell Signaling Technology, Beverly, MA, USA; 4 °C, overnight) was followed by Alexa488-conjugated secondary antibody (1:500, Jackson ImmunoResearch Laboratories, West Grove, PA, USA). Slides were mounted with Hardset Vectashield (with DAPI (4′,6-diamidino-2-phenylindole)) (Vector Laboratories, Burlington, ON, Canada) and imaged using Olympus FV10i confocal microscope.

### Protein extraction and Western blot analysis

Following the treatments with cytokines, INGAP, and/or pharmacological inhibitors of signaling pathways (Supplementary Table 2), cells were lysed in Cell Lysis Buffer (Cell Signaling Technology, Danvers, MA, USA) or radioimmunoprecipitation (RIPA) buffer (Santa Cruz Biotechnology, Dallas, TX, USA) containing 2.5 mM Na_4_P_2_O_7_, 1 mM Na_3_VO_4_, phenylmethylsulfonyl fluoride, and complete protease inhibitor cocktail tablet (Roche, Mississauga, ON, Canada) or Protease Inhibitor Mini Tablets (Pierce/Thermo Scientific, Burlington, ON, Canada) on ice. Equal amounts of protein [20–50 μg, measured with DC Protein assay (Bio-Rad)] were resolved by sodium dodecyl sulfate-polyacrylamide gel electrophoresis (SDS-PAGE) or stain-free SDS-PAGE (Bio-Rad), transferred onto nitrocellulose membrane (Bio-Rad), and probed with primary antibodies (Supplementary Table 3), secondary anti-mouse or anti-rabbit HRP-conjugated antibodies (Bio-Rad or Cell Signaling Technology), and developed using Clarity Western ECL (Bio-Rad). Chemiluminescence was acquired by ChemiDoc MP (Bio-Rad) and quantified by Image Lab software. Quantification of stain-free blots utilized total protein for normalization. Otherwise, α-tubulin was used as a loading control. Membranes were first incubated with phospho-antibodies, followed by mild stripping (0.2 M glycine, 0.1% SDS, and 0.05% Tween-20, pH 2.2) and re-probing with the corresponding non-phospho-antibodies.

Cytoplasmic and nuclear fractions were isolated using the Nuclear Protein Isolation-Translocation Assay Kit (FIVEphoton Biochemicals, San-Diego, CA, USA), following the manufacturer’s instructions, and were analyzed as above. In addition to total lane protein (stain-free gels), α-tubulin and nuclear matrix protein p84 were used as respective loading controls.

### Statistical analysis

Experiments were repeated three to eight times. The sample size (indicated in Figure legends for each set of data) was guided by the variation of effects and the statistical significance between groups. Results are expressed as means ± SEM of biological replicates. The data were normalized to control values (PBS or cytokines) to determine fold change. To determine data normality for each experiment, residual QQ plots were generated on R (Foundation for Statistical Computing). Data were considered normally distributed when points aligned strongly with the QQ line. Data were analyzed either by one-way analysis of variance (ANOVA) followed by Bonferroni post hoc test, two-way ANOVA followed by Bonferroni post hoc test or multiple *t* test comparisons corrected by Sidak–Bonferroni method. All analyses were performed using Prism 6.0 (GraphPad) software or Microsoft Excel. A *P* value < 0.05 was considered statistically significant for all comparisons.

## Results

### rINGAP and Ingap-p protect β cells against cytokine-mediated cell death

**Fig. 1:**
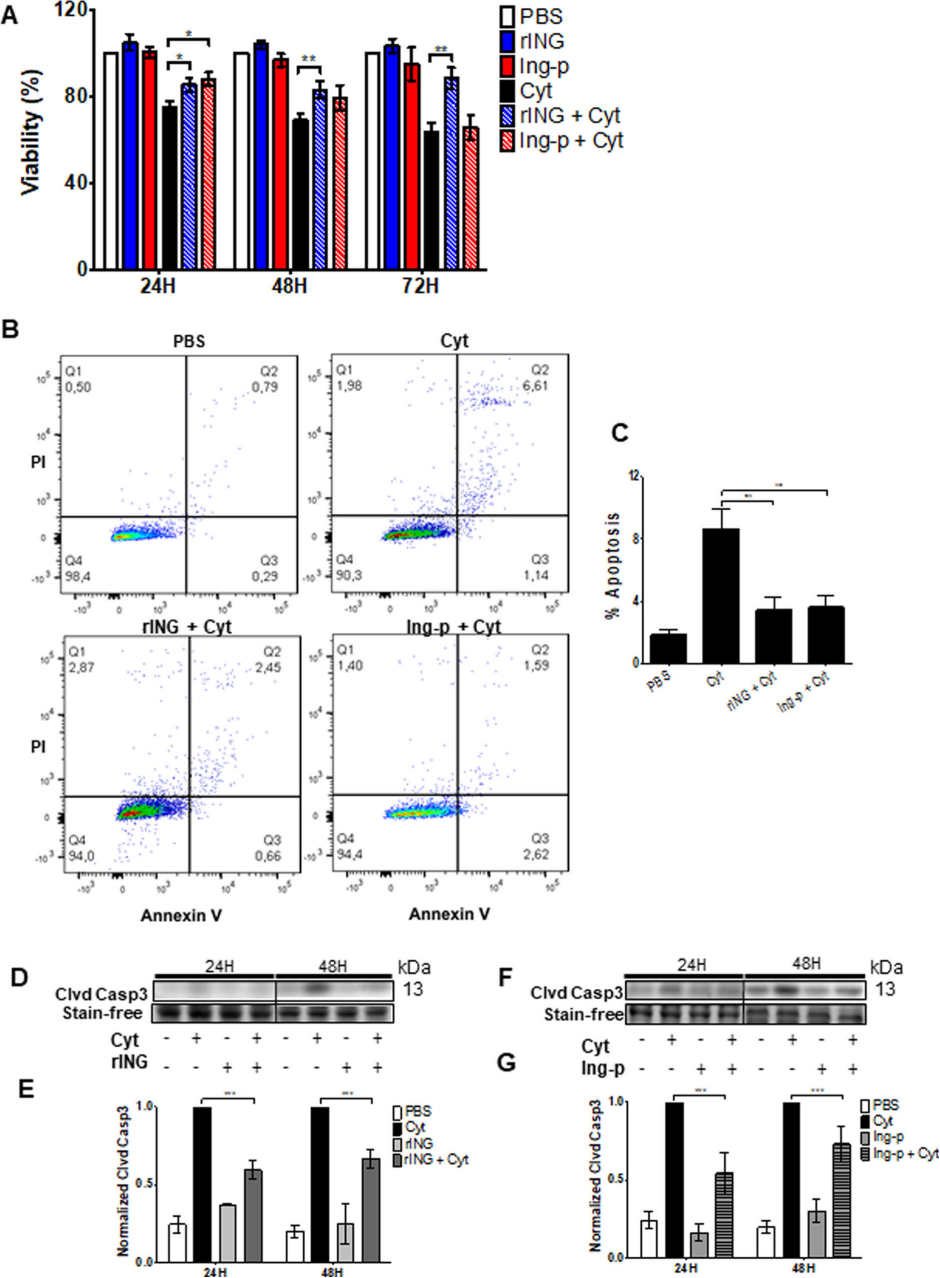
INGAP pre-treatment partially inhibits cytokine cytotoxicity in RINm5F β cells. Cells were pre-treated for 2 h with 1 nM rINGAP or 1.67 μM Ingap-p and exposed to cytokines 100 pg/mL IL-1β and 1 ng/mL IFNγ. **A** Metabolic activity was assessed by MTT assay after 24, 48, and 72 h of cytokine treatment. Values were normalized to vehicle control (PBS) and expressed as fold change. All data represent the mean of at least three independent experiments ± SEM *p < 0.05 and **p < 0.01 vs. time-matched treatment with cytokine alone; multiple *t* test comparisons corrected by the Sidak–Bonferroni method. Here and thereafter rINGAP and Ingap-p are shortened to rING and Ing-p, respectively. **B**, **C** Assessment of apoptosis by Annexin-V/PI staining after 48 h of cytokine treatment. **B** Representative dot plots of FITC-fluorescence (*x* axis) versus PI-fluorescence (*y* axis) are shown. Quadrant representation: lower left, live cells; lower right, early apoptotic cells; upper left, necrotic cells; upper right, late apoptotic and necrotic cells. **C** Percentage of total apoptotic cells was determined by summation of lower and upper right quadrants and represented as bar graphs. ***P < 0.001 vs. treatment with cytokine alone; one-way ANOVA with Bonferroni corrected multiple comparisons. **D**–**G** Level of caspase-3 assessed by Western blot. **D**, **F** Representative stain-free blots of cell lysates after 24 and 48 h cytokine treatment. **E**, **G** Densitometric analysis presented as relative quantities of cleaved caspase-3 normalized to total lane protein and expressed as fold change. All data represent the mean of at least three independent experiments ± SEM *p < 0.05 and ***p < 0.001 vs. time-matched treatment with cytokines only; multiple *t* test comparisons corrected by the Sidak–Bonferroni method.

We first pre-treated cells with 1 nM rINGAP or 1.67 μM Ingap-p for 2 h prior to cytokines and measured their viability, apoptosis, and NO production after 24, 48, or 72 h. Most data were obtained in RINm5F cells, using INS-1 cells to confirm some findings. rINGAP significantly improved viability in RINm5F cells (MTT assay, Fig. 1a), and in INS-1 cells (Supplementary Fig. 2A), at all time points, whereas Ingap-p had significant effect only at 24 h (Fig. 1a). Both rINGAP and Ingap-p reduced apoptosis, as assessed by Annexin-V-FITC/PI reactivity after 48 h in RINm5F (Fig. 1b, c) and INS-1 cells (Supplementary Fig. 2B, C), and by the cleaved Caspase-3 immunoblotting (RINm5F cells, 24 h and 48 h, Fig. 1d–g) and immunostaining in both cell lines (Supplementary Fig. 2D, E and 3C, D). This was further corroborated by the decrease in TUNEL-positive cells (47 ± 2.6% (*p* < 0.001)) and cleaved PARP in RINm5F cells by rINGAP (Supplementary Fig. 3A, B and E, F).

**Fig. 2:**
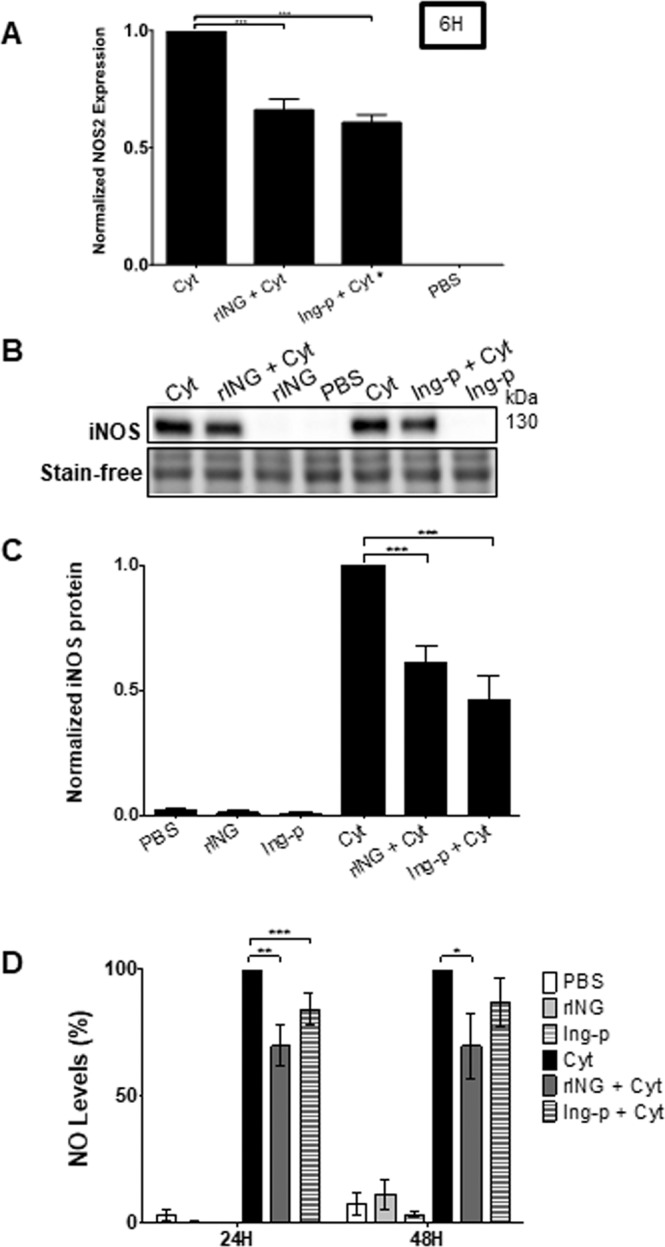
INGAP pre-treatment decreases cytokine-induced Nos2 expression and NO production in RINm5F cells. RINm5F cells were pre-treated with 1 nM rINGAP or 1.67 μM Ingap-p for 2 h prior to exposure to cytokines for 6 h. **A** Nos2 mRNA was assessed by qRT-PCR using the ΔΔCt method and normalized to cytokines only (=1). **B**, **C** Assessment of iNOS protein by Western blot: **B** Representative stain-free blots of cell lysates after 6 h cytokine exposure; **C** densitometric analysis presented as relative quantities of iNOS normalized to total lane protein and expressed as fold change. **D** NO levels in culture medium were assessed by Griess assay after 24 and 48 h. Data were normalized to cytokines only treatment and expressed as a percentage. *P < 0.05, **p < 0.01, ***p < 0.001 vs. treatment with cytokines only; **A**, **C** one-way ANOVA with Bonferroni corrected multiple comparisons; **D** two-way ANOVA with Bonferroni corrected multiple comparisons. Data represent the mean of at least three independent experiments ± SEM.

Both rINGAP and Ingap-p downregulated the cytokine-induced expression of iNOS mRNA (Nos2) and protein in RINm5F (Fig. 2a–c) and INS-1 cells (Supplementary Fig. 4A, B) and reduced NO production (Fig. 2d), although rINGAP had a longer-lasting effect (up to 48 h) than Ingap-p. Together, these data show that both rINGAP and Ingap-p partially inhibit cytokine-induced apoptosis in β cells and that this correlates with inhibition of Caspase-3, iNOS expression, and NO production.

**Fig. 3:**
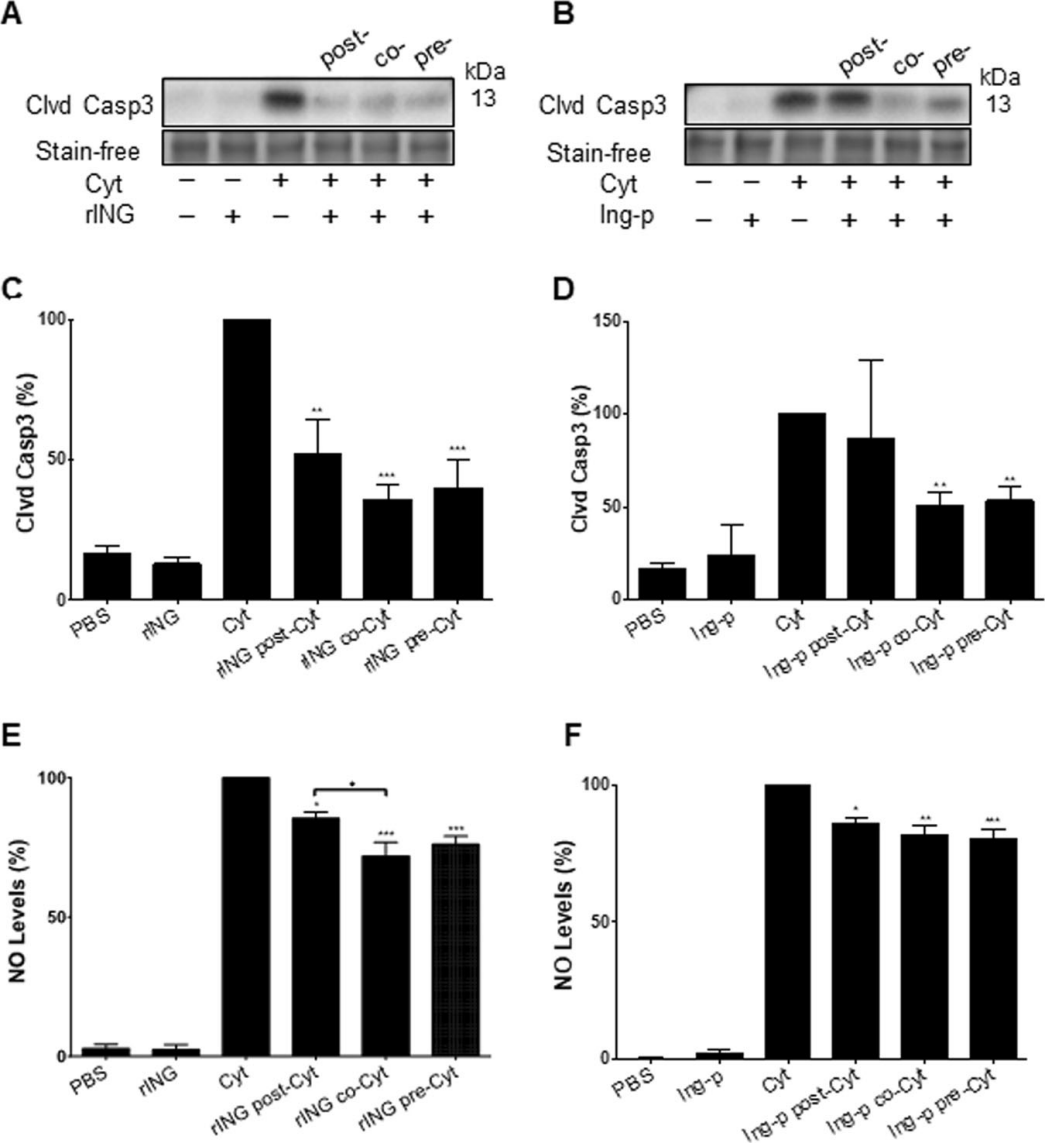
Effects of INGAP administration timing on cytokine-induced β-cell death. 1 nM rINGAP or 1.67 μM Ingap-p were administered: 2 h pre- or post-cytokines, or simultaneously with cytokines (co-treatment); **A**–**D** levels of cytokine-induced cleaved caspase-3 as a measure of apoptosis were determined by Western blot. **A**, **B** Shown are representative stain-free blots of cell lysates treated with rINGAP and Ingap-p, respectively, and **C**, **D** densitometric analysis presented as relative quantities of cleaved caspase-3 normalized to total lane protein and expressed as a percentage. *P < 0.05, **p < 0.01, and ***p < 0.001 vs. treatment with cytokines only; one-way ANOVA with Bonferroni corrected multiple comparisons and multiple *t* test comparisons corrected by the Sidak–Bonferroni method. **E**, **F** Levels of nitric oxide production were measured by Griess assay in RINm5F cells after 48 h cytokine exposure when **E** timing of 1 nM rINGAP or **F** timing of Ingap-p was varied. Values were normalized to cytokines only and expressed as a percentage. **P < 0.01 and ***p < 0.001 vs. treatment with cytokine alone; & ♦*p* < 0.05 vs. treatment with rINGAP post cytokines; one-way ANOVA with Bonferroni corrected multiple comparisons and multiple *t* test comparisons corrected by the Sidak–Bonferroni method. All data represent the mean of at least three independent experiments ± SEM.

We determined next whether the timing of INGAP administration relative to cytokine treatment (±2 h) had an impact on its anti-apoptotic effect. rINGAP reduced cleaved caspase-3 when administered either pre-, co-, or post-cytokine treatment (Fig. 3a, c), whereas Ingap-p was effective in pre- and co- but not post-cytokine treatment (Fig. 3b, d). NO production after 48 h was significantly reduced by rINGAP and Ingap-p in all groups, however, rINGAP was more effective as co- and pre-treatment (Fig. 3e, f). We chose the pre-treatment mode to characterize the signaling mechanism by which rINGAP and Ingap-p elicit anti-apoptotic effects.

### INGAP targets cytokine-induced NF-κB signaling by inhibiting p65 phosphorylation

In keeping with previous reports^41,48,49^, cytokines activated several signaling pathways, most notably NF-κB, JAK-STAT, p38, and JNK (Supplementary Fig. 5). Inhibition of these pathways, particularly NF-κB and JAK-STAT, significantly reduced iNOS, while inhibition of p38 and JNK was less effective (Supplementary Fig. 6A–D). This suggests that NF-κB and JAK/STAT signaling play predominant roles in cytokine-induced cytotoxicity, whereas p38 and JNK are secondary.

NF-κB signaling involves phosphorylation of the IKKα/β and IκBα, leading to IκBα ubiquitination and proteosomal degradation. This allows the NF-κB heterodimer p65(RelA)/p50 to translocate to the nucleus and modulate transcription^50,51^. Transcriptional activity, stability, and proteosomal degradation of NF-κB are believed to depend on p65 phosphorylation^52^.

**Fig. 4:**
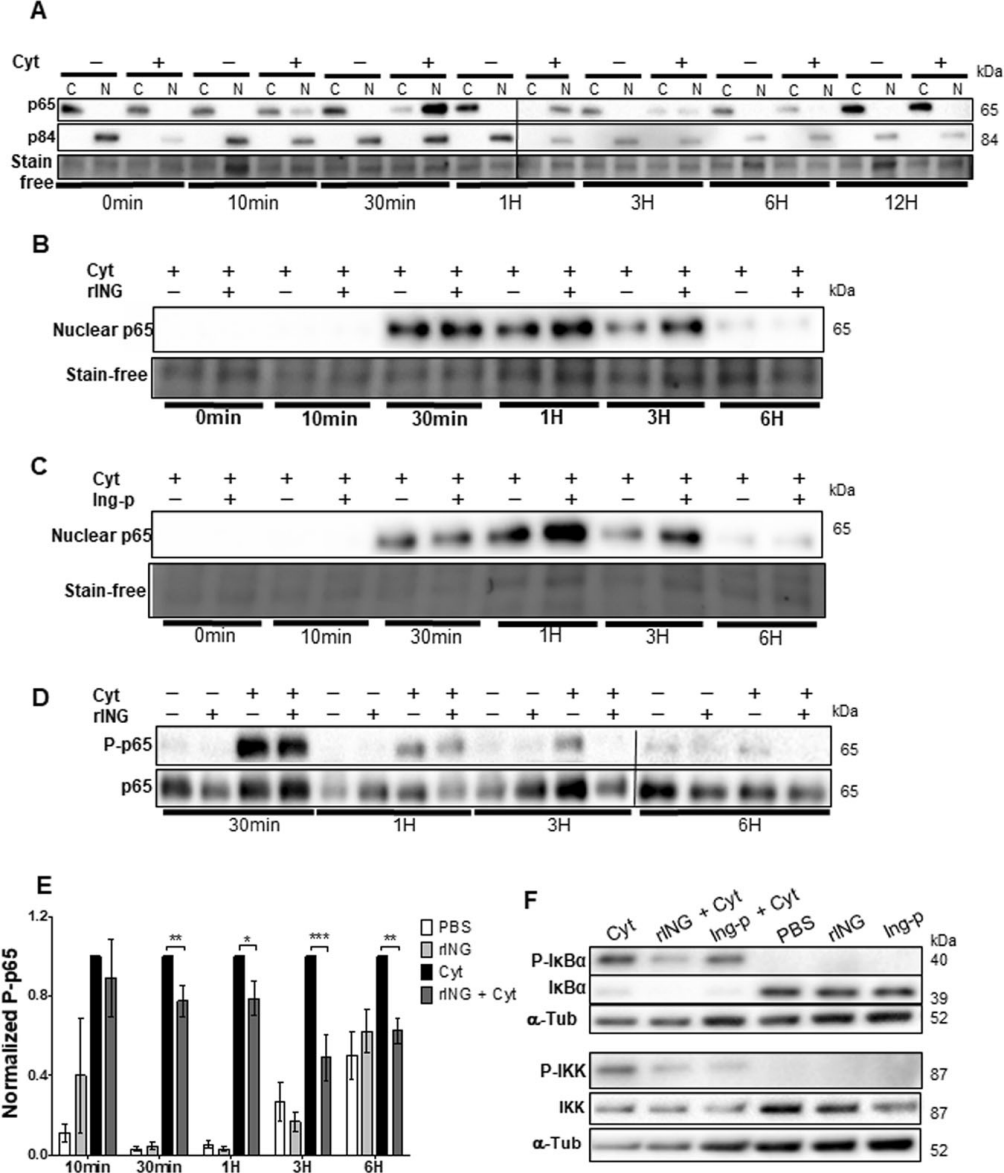
Effect of INGAP pre-treatment on cytokine-induced p65 nuclear translocation and phosphorylation. RINm5F cells were treated with cytokines ± INGAP in the time-course experiments followed by extraction of cytoplasmic (C) and nuclear (N) proteins **A**–**C** or total proteins **D**–**F** and analyzed by Western blot. Shown are representative stain-free blots of subcellular lysates from cells treated with cytokines compared with **A** PBS; **B** rINGAP; and **C** Ingap-p. p84 was included as a loading control for nuclear fractions and total protein was used as a loading control for all samples. **D** p65 phosphorylation (P-p65) in cell lysates during time-course treatment of 6 h. **E** Densitometric analysis of each time point; values presented as P-p65 normalized to total lane protein and expressed as fold change over cytokines only. Data expressed as means ± SEM *P < 0.05, **p < 0.01, ***p < 0.001 vs time-matched cytokine cocktail; multiple t test comparisons corrected by the Sidak–Bonferroni method. **F** Analysis of IKK and IκBα phosphorylation after 1 h exposure to cytokines. Tubulin was used as a loading control. All data are representations from three independent experiments.

NF-κB activation by cytokines in RINm5F cells induced rapid and transient phosphorylation of IKK ((Ser^176/180^) 10 min–1 h, peak at 30 min), and IκBα ((Ser^32^) 10 min–1 h), accompanied by p65 phosphorylation at Ser^536^ (10 min–6 h, Supplementary Fig. 5A) and p65 nuclear translocation (10 min–3 h, peak at 30 min, Fig. 4a). The peak in P-p65 was at 10 min, preceding that in P-IKK and P-IκBα. This fact, together with limited inhibition by IKKVII (Supplementary Fig. 6E), suggests that p65 phosphorylation was at least partially IKK-independent.

rINGAP and Ingap-p decreased the cytokine-induced phosphorylation of IKK and IkBα (Fig. 4f) but did not inhibit p65 nuclear translocation, which is necessary for exerting the effects of NF-κB signaling on the target genes. In contrary, nuclear p65 was increased in INGAP pre-treated cells (Fig. 4b, c). The cause of this is unclear since rINGAP itself does not induce p65 translocation (data not shown). However, rINGAP significantly decreased p65 phosphorylation at Ser^536^ between 30 min and 6 h of cytokine treatment (Fig. 4d, e). Same effect was observed for Ingap-p at 6 h (Supplementary Fig. 7A, B). These results suggest that INGAP may affect the NF-κB signaling through p65 phosphorylation, potentially modulating p65 transcriptional activity or stability. Interestingly, inhibition of P-p65 (Ser^536^) was further increased when rINGAP was used together with IKKVII inhibitor (Supplementary Fig. 7C, D), suggesting that besides IKK, INGAP may target other kinases implicated in p65 phosphorylation at Ser^536^ ^53^.

### Effects of rINGAP on NF-κB signaling is observed when cytokines are used together but not separately

**Fig. 5:**
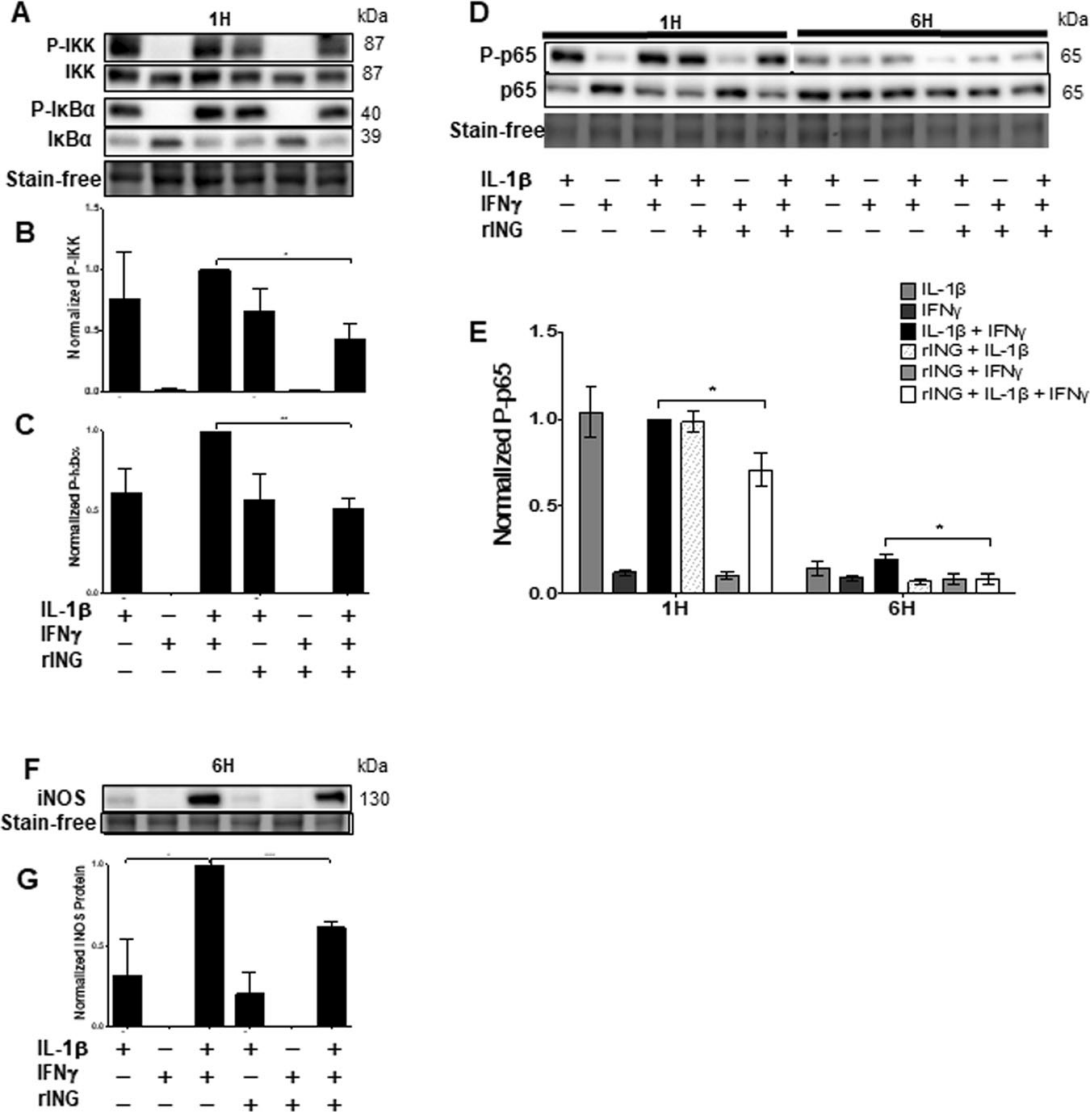
rINGAP pre-treatment inhibits NF-κB signaling when IL-1β and IFNγ used together but not individually. RINm5F cells were pre-treated with 1 nM rINGAP for 2 h and then exposed to 100 pg/mL IL-1β, 1 ng/mL IFNγ, or a cocktail of both. Equal amounts of protein were resolved on stain-free SDS-PAGE and analyzed by Western blot to assess changes in: **A** phospho(P)-IKK, and phospho(P)-IκBα, with densitometric analysis shown in **B** and **C**, respectively; **D**, **E** phospo(P)-p65 and **F**, **G** iNOS. Shown are representative blots from three independent experiments. Densitometric data in **B**, **C** and **E** are presented as relative quantities of phosphorylated/total protein ratio normalized to total lane protein and expressed as fold change of cytokine cocktail. **G** Values for iNOS were normalized by total lane protein and are shown as fold change of cytokines. Data expressed as means ± SEM *P < 0.05, **p < 0.01, ***p < 0.001 vs time-matched cytokine cocktail; multiple *t* test comparisons corrected by the Sidak–Bonferroni method.

To better understand the impact of rINGAP on NF-κB signaling, we repeated the experiment with each cytokine separately. As expected, NF-κB pathway was induced by IL-1β but not by IFNγ, which, however, potentiated the effects of IL-1β on phosphorylation of IKK and IκBα (Fig. 5a–c), and on iNOS expression (Fig. 5e). p65 phosphorylation was not enhanced by IFNγ (Fig. 5d), indicating that it was under control of IL-1β only. Interestingly, all inhibitory effects of rINGAP were only observed for the cytokine cocktail but not IL-1β alone, even for p65. In the case of IKK, IκBα, and iNOS, the decreases were roughly equal to reversing the potentiation by IFNγ (Fig. 5a–c, e). This suggests that INGAP targeted the IFNγ signaling rather than IL-1β.

### INGAP is more effective against the cytokine cocktail than individual cytokines in inhibiting STAT3, p38, and JNK

**Fig. 6:**
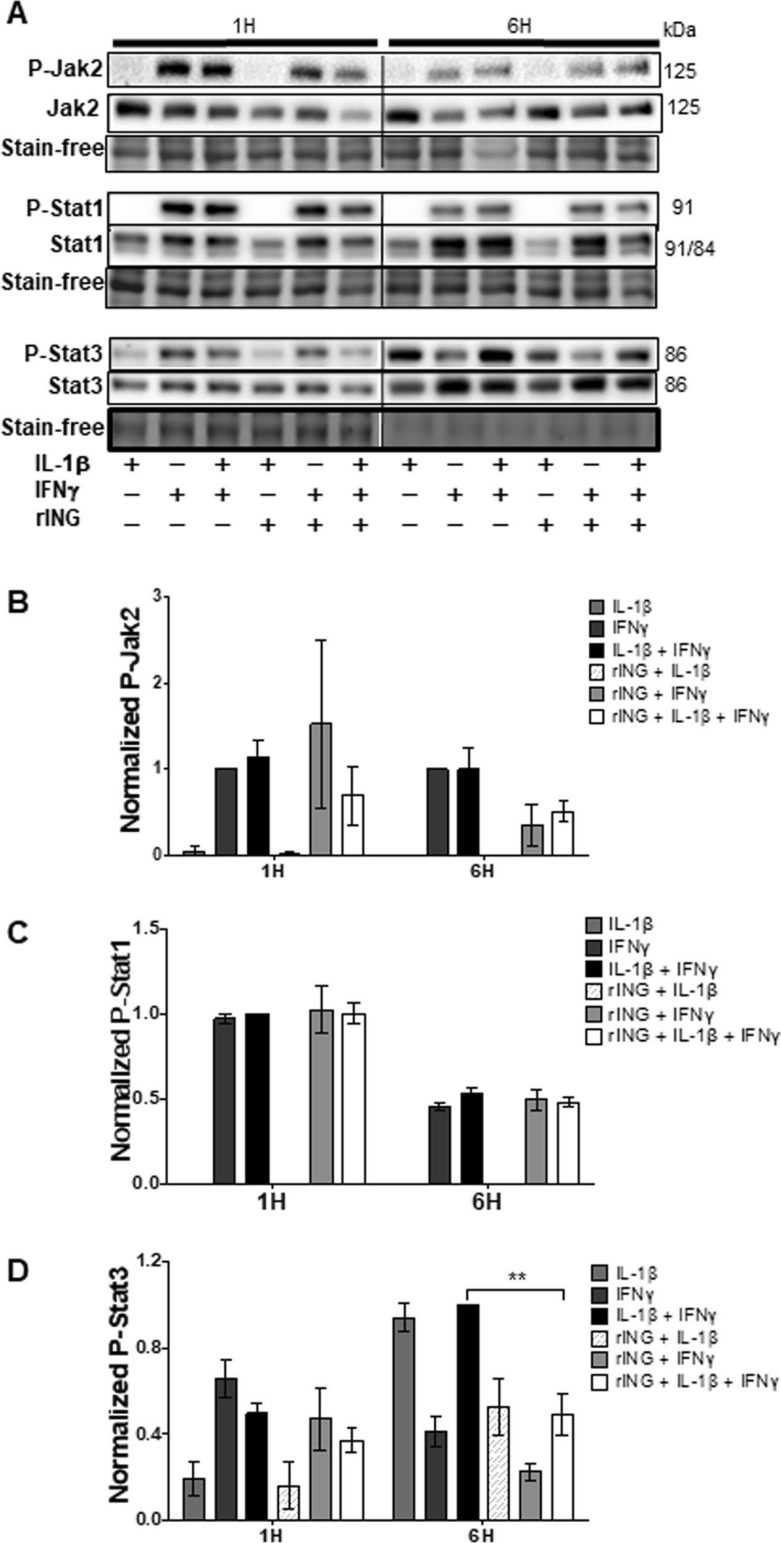
Effects of rINGAP on Jak2/Stat1/Stat3 phosphorylation in RINm5F treated with cytokines IL-1β, IFNγ individually, and in combination. RINm5F cells were pre-treated with 1 nM rINGAP for 2 h and then exposed to 100 pg/mL IL-1β, 1 ng/mL IFNγ, or a cocktail of both for 1 or 6 h. Equal amounts of protein were resolved on stain-free SDS-PAGE and Western blot analysis was used to assess changes in phosphorylation of Jak2, Stat1, and Stat3. **A** Shown are representative blots from three independent experiments and **B–D** densitometric analysis presented as relative quantities of phosphorylated/total protein ratio normalized to total lane protein and expressed as fold change of cytokines only. Values expressed as means ± SEM **P < 0.01 vs time-matched cytokine cocktail; multiple *t* test comparisons corrected by the Sidak–Bonferroni method.

JAK2/STAT1 signaling was activated by IFNγ, resulting in phosphorylation of JAK2 (Tyr^1007/1008^), and STAT1 (Tyr^701^) at 1 h and 6 h, whereas IL-1β had no effect. In contrast, STAT3 phosphorylation (Tyr^705^), although induced mostly by IFNγ at 1 h, was driven mostly by IL-1β at 6 h (Fig. 6a). Used in combination, cytokines increased P-STAT3 up to 12 h of treatment (Supplementary Fig. 5B). rINGAP had no effect on STAT1 phosphorylation (Fig. 6a, c) but slightly decreased P-JAK2 induced by IFNγ or both cytokines at 6 h (Fig. 6a, b). It also inhibited P-STAT3 at 1 h and 6 h (all combinations, Fig. 6a, d), but the differences were statistically significant only for both cytokines at 6 h, corroborating that rINGAP was more potent against the cytokine cocktail.

**Fig. 7:**
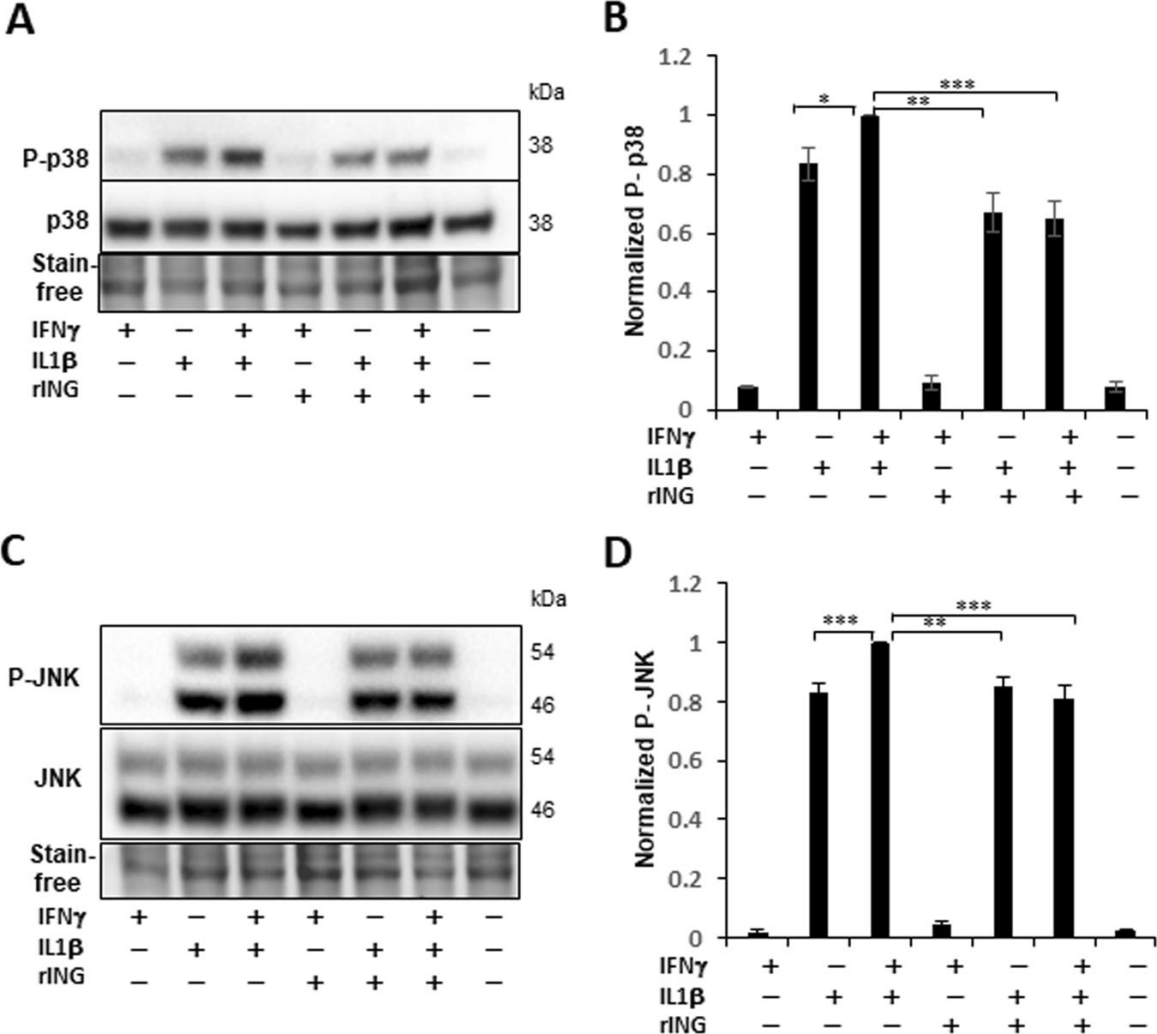
rINGAP pre-treatment inhibits p38 and JNK activation when IL-1β used with IFNγ but not alone. RINm5F cells were pre-treated with 1 nM rINGAP for 2 h and exposed to cytokines for 30 min. Lysates were resolved on stain-free SDS-PAGE and probed for: **A**, **B** P-(phospho)-p38 and **C**, **D** P-JNK followed by pan- p38 and JNK antibodies. **A, C** Shown are representative blots of eight experiments and **B, D** the results of densitometric analysis shown as a ratio of phospho/total protein relative to the cytokine cocktail. *P < 0.05, **p < 0.005, ***p < 0.001 (*t* test).

Phosphorylation of JNK (Thr^183^/Tyr^185^) and p38 (Thr^180^/Tyr^182^), most prominent at 30 min (Supplementary Fig. 5B), was induced by IL-1β but not by IFNγ, which, however, potentiated the IL-1β effects (Fig. 7). rINGAP pre-treatment inhibited both kinases and, in keeping with earlier results, this was observed when IL-1β was used with IFNγ but not alone and was roughly equal to eliminating the effect of IFNγ.

### rINGAP downregulates expression of IFNγ-responsive genes induced by cytokine cocktail

**Fig. 8:**
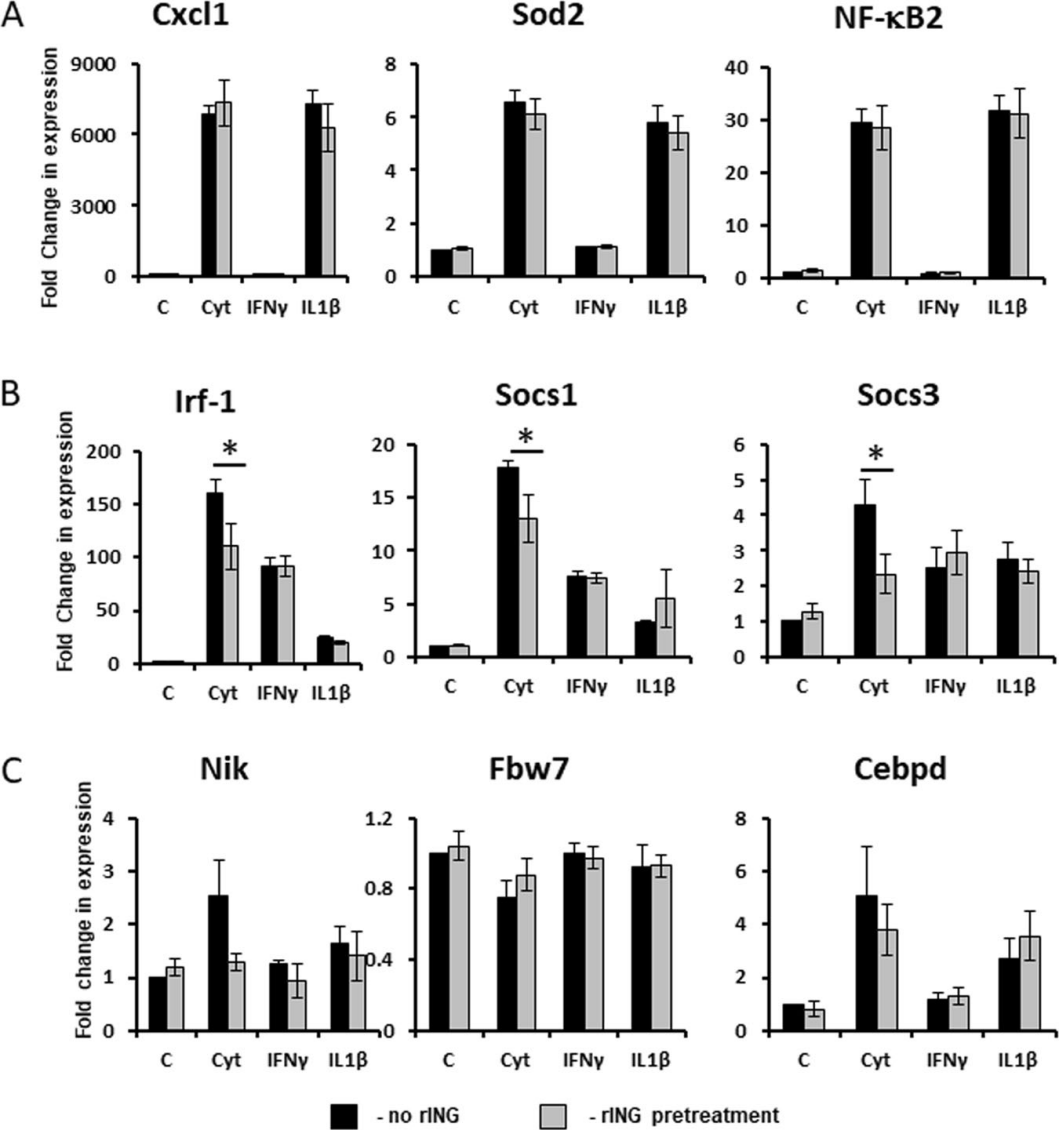
Effect of rINGAP on the expression of cytokine-stimulated genes. Rinm5F cells were pre-treated with 1 nM rINGAP for 2 h and treated with cytokines, individually or in combination for 3 h and processed for RNA extraction and qRT-PCR. Experiments were repeated five times. Fold change in gene expression over control (PBS) was calculated with the CFX Manager Software 3.1 using three reference genes (Gapdh, β-actin, and α-tubulin). Target stability was within the range M < 0.5, CV < 0.25. *P < 0.05 vs PBS control: *t* test one-tailed, two-sample equal variance.

Since rINGAP did not inhibit p65 nuclear translocation or JAK2/STAT1 phosphorylation, we examined next whether it affected the expression of the downstream target genes. We chose several known IL-1β/NF-κB-stimulated^40,41^ and IFNγ-stimulated genes^42,54^ involved in β-cell apoptosis for analysis by qRT-PCR. Based on cytokine responsiveness, selected genes were categorized into: (1) IL-1β-induced (e.g., NF-κB2, Sod2, Cxcl1); (2) IL-1β-induced and IFNγ-potentiated (Nos2); (3) predominantly IFNγ-induced (Irf1, Socs1, Socs3); and (4) non-canonical NF-κB associated genes (Fbw7, Cebpd, Nik) (Supplementary Table 1, Fig. 8). Although the time of induction differed among these genes, at 3 h post cytokines all genes were elevated, favoring this time point for rINGAP experiments. In keeping with the lack of effect on NF-κB, rINGAP had no effect on the IL-1β-induced genes but downregulated the IFNγ-induced genes (Fig. 8a, b). However, once again, the effect was observed only with both cytokines and not IFNγ alone. Among the genes involved in regulation of non-canonical NF-κB pathway, Nik (NF-κB inducing kinase) appeared also downregulated by rINGAP, albeit the change was not statistically significant. Likewise, only a trend and no significant change was shown for Fbw7 and Cebpd^40^ (Fig. 8c).

## Discussion

This is the first study to demonstrate the anti-apoptotic effects of recombinant INGAP and INGAP-peptide in the cytokine-treated β cells. Although pro-survival effects of INGAP have been reported^29,33^, the impact of pro-inflammatory conditions was not evaluated. In this work, we used a common model of cytokine-induced death that employs IL-1β and IFNγ, two cytokines most implicated in the development of diabetes^5,6^. In contrast to other studies, we also used each cytokine separately to better understand the underlying signaling events. Our main findings are: (1) both rINGAP and Ingap-p reduced cytokine-induced apoptosis, which correlated with inhibition of cleaved Caspase-3 and of the iNOS-NO axis; (2) rINGAP inhibited phosphorylation of p38, JNK, STAT3, and the constituents of NF-κB pathway, whereas nuclear translocation of NF-κB and the downstream gene expression was not inhibited; (3) rINGAP was more effective against the cytokine cocktail than each cytokine separately and appeared to reverse the potentiating effects of IFNγ; (4) expression of IFNγ-stimulated genes was downregulated by rINGAP when cytokines were used in combination but not IFNγ alone; (5) taken together, our data suggest that rINGAP affects the crosstalk between IL-1β and IFNγ signaling.

In keeping with our previous research^16,17^, we compared the effects of rINGAP and Ingap-p. Ingap-p, shown to reproduce the effects of the INGAP protein^11^, has been used in multiple studies worldwide^22–29^ including clinical trials^19^. The interest in Ingap-p is obvious because it is synthetic and well-tolerated by patients^13,19^. However, as we have previously shown, rINGAP is more stable and over 100× more potent on a molar basis than Ingap-p, and it interacts differently with the cell surface, which may potentially lead to different outcomes ^11,16,17^. Here again, we demonstrate a stronger potency for rINGAP versus Ingap-p in inhibiting apoptosis, improving viability (72 h vs 24 h), and reducing NO production (48 h vs 24 h). Importantly, rINGAP demonstrated a “rescue” effect when administered 2 h post cytokines, whereas Ingap-p was effective only in pre- and co-treatment modes. Whereas a greater stability of rINGAP could account for a stronger improvement in viability, the “rescue” effect clearly points to some differences in the mechanism of action. These differences would be interesting to explore to potentially design a better peptide for future clinical use.

Among signaling pathways involved in cytokine-induced β-cell death, NF-κB plays a major role^55^, exerted mainly through activation of iNOS expression and NO production. Although cytokines may act in a NO-independent fashion^56^, the critical role of NO in IL-1β/NF-κB-induced β-cell dysfunction and apoptosis has been well established^46,57–59^. Inhibition of NF-κB signaling leads to cytoplasmic retention of p65 that halts its transcriptional activity^60^. As we showed, although rINGAP and Ingap-p decreased IL-1β + IFNγ-induced phosphorylation of IKK, IκBα, and p65, p65 nuclear translocation was not inhibited and even slightly enhanced. This result was unexpected, as it somewhat contradicted the inhibitory effects of INGAP on iNOS expression/NO production and on the aforementioned phosphorylation events. We then investigated by qRT-PCR the expression of several NF-κB-regulated chemokine genes^40,61^ to test whether rINGAP inhibited the p65 transcriptional activity. Expression of NF-κB2, Sod2, Cxcl1 shown in Fig. 8a, plus a few others, such as Ccl2, Lcn2, Ccl5, Ccl19 (data not shown) upregulated by IL-1β ± IFNγ, but not by IFNγ, was not reduced by rINGAP. We, therefore, concluded that rINGAP did not inhibit NF-κB signaling.

The significance of the INGAP effect on p65 phosphorylation is less clear, as the role of phosphorylation in the IκBα-regulated p65 translocation and transcriptional activity is poorly understood. Aside from the IKK complex (mostly IKKβ)^52,62^, several kinases such as RSK1, MSK1, TAK1 have been shown to phosphorylate p65 on Ser^536^ ^52^. There is evidence that phosphorylation may promote p65 disassociation from IκBα and p50, which carries a dominant nuclear export sequence^63,64^, causing a delayed nuclear translocation^65^ or an increase in p65 turnover^66^. It is possible, therefore, that inhibition of p65 phosphorylation by INGAP contributed to accumulation and/or a delayed degradation of nuclear p65 observed in this study.

We show that p65 phosphorylation is induced by IL-1β and is not affected by IFNγ. It is not clear, however, why rINGAP inhibited p65 phosphorylation only when IL-1β was used together with IFNγ but not alone. It is possible that rINGAP and IFNγ synergistically inhibit one of the kinases that phosphorylate p65, whereas such a synergy is absent between INGAP and IL-1β but to verify this possibility a more-detailed investigation into the signaling pathways of IFNγ and rINGAP is needed.

IFNγ is often used with IL-1β to model the diabetic milieu, although its role in β-cell apoptosis and the signaling pathways leading to it are less understood. We show that IFNγ had no effect on the NF-κB-iNOS-NO axis but potentiated the action of IL-1β on the phosphorylation of IKK, IκBα, and significantly increased iNos expression. As rINGAP eliminated the IFNγ-potentiating effects, we reasoned that it targeted IFNγ signaling and screened for known IFNγ-responsive genes^54^. Of note, out of several genes tried, only three—Irf1, Socs1, and Socs3, were induced by IFNγ more than by IL-1β. We show that rINGAP indeed reduced the expression of these genes but, once again, for the cocktail of both cytokines and not IFNγ alone. rINGAP did not inhibit STAT1 phosphorylation, which is required for IFNγ signaling^67^. This suggests that INGAP targets neither IFNγ nor IL-1β specifically but modulates the crosstalk between the two, the precise mechanisms of which are yet to be elucidated.

Cytokine signaling involves multiple pathways. IL-1β has been shown to activate several signaling pathways including NF-κB, JAK/STAT, PI3K, and MAPK^40,41,48^. Likewise, IFNγ-induced signaling, previously thought to be limited to JAK/STAT1 pathway, has been expanded to include STAT3, STAT5, Src family of non-receptor tyrosine kinases, PI3K, NF-κB, MAP kinases that are recruited by IFNγR or JAK^42,68^. These pathways constitute a complex network that may account for multiple effects elicited by IFNγ alone, and in combination with IL-1β^45,46,67^. This complexity makes it difficult for us to pinpoint the possible target of INGAP, as we addressed only the most common pathways, such as NF-κB, JAK/STAT, and MAPK. However, this is the first step that builds the base for further investigation.

In addition to the complexity of cytokine crosstalk, INGAP signaling is not well understood either. No specific receptor has been identified and the existing studies report a wide variety of INGAP-activated pathways, such as PI3K/Akt^15,17,26^, cholinergic pathway^26^, NF-κB^69^, or cAMP-PKA^70^. We have previously shown that rINGAP and Ingap-p stimulate proliferation in RINm5F cells by activating Ras-Raf-Erk1/2 pathway, which may also involve Src^17^. However, we did not observe activation of NF-κB, PKA, p38 MAPK, or PKC in RINm5F cells by either rINGAP or Ingap-p, which is perhaps cell type specific. The search for possible mechanisms by which INGAP exerts its anti-cytokine effects might be perhaps narrowed to the Ras-Raf-Erk1/2 and PI3K/Akt pathways, which should be investigated in more detail.

In summary, INGAP has been known for its regenerative properties and the ability to reverse diabetes in animal models^10,13,15,18,20,71^. Our work shows that INGAP also protects β cells from cytokine-induced apoptosis, thus providing new insights into the anti-diabetic potential of INGAP. The significance of these findings is determined by the need to develop β-cell survival therapies for the protection of the existing or transplanted β cells in the pro-inflammatory diabetic milieu. Although this study did not propose a definite mechanism of anti-apoptotic action of INGAP, it built the foundation for further research that will also include human islets and other cytokines, e.g., TNFα, to better approximate the diabetic milieu and to better evaluate the therapeutic potential of INGAP.

## Supplementary information

Supplementary Table 1

Supplementary Table 2

Supplementary Table 3

Supplementary Figure Legends

Supplementary Fig. 1

Supplementary Fig. 2

Supplementary Fig. 3

Supplementary Fig. 4

Supplementary Fig. 5

Supplementary Fig. 6

Supplementary Fig. 7
