# Supplementary material for: Islet neogenesis associated protein (INGAP) protects pancreatic β cells from IL-1β and IFNγ-induced apoptosis
Source: Cell Death Discov. 2021 Mar 17;7:56. doi: 10.1038/s41420-021-00441-z (PMC7969959; doi:10.1038/s41420-021-00441-z)
Supplement: Supplementary file 1 — Supplementary Table 1 [file 41420_2021_441_MOESM1_ESM.docx]

**Supplementary Table 1. Primers used in the study**

|  | | | | |
| --- | --- | --- | --- | --- |
| **Gene** | **Reference** | **Forward** | **Reverse** | **Amplicon (bp)** |
|  |  | **Reference genes** |  |  |
| β-Actin | NM_031144.2 | CCGTAAAGACCTCTATGCCAAC | CTCAGGAGGAGCAATGATCTTGAT | 135 |
| Gapdh | NM_017008.3 | CTGGAGAAACCTGCCAAGTATGATG | GGAAGAGTGGGAGTTGCTGTTGA | 134 |
| α-Tub | NM_022298.1 | ATTCGCAAGCTGGCTGACCA | TGTGGGTGGTGAGGATGGA | 213 |
|  |  | **IL-1β-stimulated genes** |  |  |
| Ccl2 | NM_031530.1 | CTGTCTCAGCCAGATGCAGTT | ACTTCTGGACCCATTCCTTATTGG | 192 |
| Ccl5 | NM_031116.3 | CCTTGCAGTCGTCTTTGTCACTC | CAGGATCAGAATGGAGAGACCCTC | 127 |
| Ccl19 | NM_001108661.1 | TTCCTCCAAGAGCAAAGGCG | ACTCACGTTCACACCGACTCT | 172 |
| Cxcl1 | NM_030845.1 | CCGAAGTCATAGCCACACTCAAGA | GTTGTCAGAAGCCAGCGTTCA | 167 |
| Nos2 | NM_012611.3 | CTCCCCATTCTGAAGCCC | ACTGACACTCCGCACAAAG | 197 |
| Fas | NM_139194.2 | CCGTGTCAGCCTGGTGAACGAAA | CCACTTCTAAACCATGCCCTTCATC | 160 |
| Sod2 | NM_017051.2 | AGAACCCAAAGGAGAGTTGCTGG | CTTGCAGTGGGTCCTGATTAGAGC | 181 |
|  |  | **IFNγ-stimulated genes** |  |  |
| Irf-1 | NM_012591.1 | CAAGAGGAAGCTGTGCGGAGAT | AGAGAGACTGCTGCTGACGAC | 177 |
| Socs1* | NM_145879.2 | CACTTCTGGCTGGAGACCTTATCC | TGGAGAGGTAGGCGTGGAGTT | 125 |
| Socs3* | NM_053565.1 | TTCTCTTTACCACCGACGGAACC | CTTTCTCATAGGAGTCCAGGTGACC | 209 |
|  |  | **Non-canonical Nf-κB** |  |  |
| Nf-κB2 | NM_001008349.1 | GGCAGTCTCCTTCGTAGTTACAAGC | TCTTCTTTCACCTCTGTGCTGGG | 148 |
| Nik | NM_001108301.1 | TCAGCATCGACAGCCTCTCA | GACCTTGACACCGTTGAAGTAGC | 196 |
| Fbxw7 | XM_002729089.5 | GCAGGAGGGTTGTTAGTGGAGC | TGCCGTCAAACTGTAACGAATAGAC | 122 |
| Cebpd* | NM_013154.2 | GCCGACCTCTTCAACAGCAAT | CCAAGCTCACCACTGTCTGC | 201 |

Primers were designed to efficiently anneal at 58-60°C. Except for one-exon genes (*), at least one primer in each pair spans a splice site to exclude amplification of genomic DNA. Veracity of PCR products was confirmed by appearance of single bands of indicated sizes on 1.5% agarose gel.
