# Supplementary material for: Islet neogenesis associated protein (INGAP) protects pancreatic β cells from IL-1β and IFNγ-induced apoptosis
Source: Cell Death Discov. 2021 Mar 17;7:56. doi: 10.1038/s41420-021-00441-z (PMC7969959; doi:10.1038/s41420-021-00441-z)
Supplement: Supplementary file 2 — Supplementary Table 2 [file 41420_2021_441_MOESM2_ESM.docx]

**Supplementary Table 2**

**Pharmacological inhibitors used in the study**

| **Inhibitor** | **Targeted pathway** | **Concentration** | **Source/ Cat#** |
| --- | --- | --- | --- |
| IKK inhibitor VII | NF-κB | 1μM or 10μM | EMD Millipore/Calbiochem  #401486 |
| JAK inhibitor I | JAK/STAT | 10μM | EMD Millipore/Calbiochem  #420099 |
| AG490 | JAK2 | 100μM | EMD Millipore/Calbiochem  #658408 |
| SB202190 | p38 MAP Kinase | 1μg/mL | EMD Millipore/Calbiochem  #559397 |
| SP600125 | c-Jun N-terminal kinase (JNK) | 50μM | EMD Millipore/Calbiochem  #420119 |
