# Supplementary material for: Islet neogenesis associated protein (INGAP) protects pancreatic β cells from IL-1β and IFNγ-induced apoptosis
Source: Cell Death Discov. 2021 Mar 17;7:56. doi: 10.1038/s41420-021-00441-z (PMC7969959; doi:10.1038/s41420-021-00441-z)
Supplement: Supplementary file 3 — Supplementary Table 3 [file 41420_2021_441_MOESM3_ESM.docx]

**Supplementary Table 3**

**Primary Antibodies used in the study***

| **Phospho-Antibodies / Cat #** | **Non-Phospho Antibodies/ Cat #** | **Dilution** | **Source** |
| --- | --- | --- | --- |
| STAT1 (Tyr^701^) d4a7 /#7649 | STAT1 /#9172 | 1:1000 | Cell Signaling |
| phospho-Jak1 (Tyr^1022/1023^) /#3331 | Jak1 /#3344 | 1:1000 | Cell Signaling |
| phospho-Jak2 (Tyr^1007/1008^) /#3776 | Jak2 (D2E12) XP^®^ /#3230 | 1:1000 | Cell Signaling |
| phospho-Stat3 (Tyr^705^)/ #9145 | STAT3(D1A5) XP^®^ /#8768 | 1:1000 | Cell Signaling |
| phospho-p38 (Thr^180^/Tyr^182^) /#4511 | p38 MAPK(D13E1) XP^®^ /8690 | 1:1000 | Cell Signaling |
|  | Cleaved Caspase-3 /#9664 | 1:1000 | Cell Signaling |
|  | Cleaved PARP #5625 | 1:1000 | Cell Signaling |
| p-JNK (Thr^183^/Tyr^185^) /#sc-6254 | JNK /#sc-7345 | 1:1000 | Santa Cruz |
|  | iNOS /#PA1-036 | 1:1000 | Thermo-Fisher |
|  | Alpha-Tubulin /#ab7291 | 1:1000 | Abcam |
|  | Nuclear Matrix p84 /#ab487 | 1:1000 | Abcam |
| NF-κB Pathway Sampler kit containing phospho- and non-phospho- antibodies /#9936 | | 1:1000 | Cell Signaling |

* All phospho-antibodies were diluted in TBST with 5% BSA, whereas all non-phospho and secondary antibodies were in TBST/5% skim milk.
