# Supplementary material for: Islet neogenesis associated protein (INGAP) protects pancreatic β cells from IL-1β and IFNγ-induced apoptosis
Source: Cell Death Discov. 2021 Mar 17;7:56. doi: 10.1038/s41420-021-00441-z (PMC7969959; doi:10.1038/s41420-021-00441-z)
Supplement: Supplementary file 4 — Supplementary Figure Legends [file 41420_2021_441_MOESM4_ESM.docx]

**Supplementary Figure Legends:**

**Supplementary Figure 1: Characterization of the model of cytokine-induced** β**-cell cytotoxicity.**

RINm5F or INS-1 cells were pre-treated for 2 hours with 1 nM rINGAP or 1.67 μM Ingap-p and exposed to cytokines 100 pg/mL IL-1β and/or 1 ng/mL IFNγ. Data are shown as the means of at least 3 independent experiments ± S.E.M. **A** Nos2 gene expression was assessed in a time-course experiment by qRT-PCR. Data represent fold change in Nos2 mRNA relative to the cytokine cocktail at 6 h; ◦ represents the means of two independent experiments. **B, C** Nitric Oxide (NO) production was assessed by Griess assay in **B** RINm5F and **C** INS1 cells after 24 and 48 hours of cytokine treatment. **p < 0.01 and ***p < 0.001 vs. time-matched treatment with vehicle (PBS) alone; ◆p < 0.05, ◆◆p < 0.01, and ◆◆◆p < 0.001 vs. time-matched treatment with IL-1β; ■■p < 0.01 and ■■■p < 0.001 vs. time-matched treatment with IFNγ; two-way ANOVA with Bonferroni corrected multiple comparisons. **D** Effect of cytokines on cell viability was assessed by MTT assay after 48 h. Data are shown relative to PBS control. **E-G**: Assessment of various concentrations of the cytokine cocktail on NO production and cell viability: **E** NO measurements (Griess assay) in RINm5F cells after 24 hours. Data was normalized to 1x cytokines and expressed as a percentage. ***p < 0.001 vs. 1x cytokines; ◆◆◆p < 0.001 vs. 10x cytokines; one-way ANOVA with Bonferroni corrected multiple comparisons. ✼ represents mean ± S.E.M of 2 independent experiments. **F, G** Metabolic activity was assessed by MTT assay in **F** RINm5F and **G** INS1 cells after 24 and 48 hours exposure to cytokine cocktails. Data are presented as percentage of vehicle control (PBS) and expressed as fold change. ***p < 0.001 vs. time-matched treatment with vehicle (PBS) alone; ◆p < 0.05, ◆◆p < 0.01 and ◆◆◆p < 0.001 vs. time matched treatment with 1x cytokines; ■■■p < 0.001 vs. time matched treatment with 10x cytokines; **F** multiple t-test comparisons corrected using Sidak-Bonferroni method and **G** two-way ANOVA with Bonferroni corrected multiple comparisons.

**Supplementary Figure 2: rINGAP pre-treatment partially inhibits cytokine-induced apoptosis in INS-1** β **cells.**

Cells were pre-treated for 2 hours with 1 nM rINGAP then exposed to cytokines (100 pg/mL IL-1β and 1 ng/mL IFNγ). **A** Viability was assessed by MTT assay after 24 and 48 hours cytokine treatment. Data are expressed as percentage of vehicle control (PBS). *p < 0.05 and ***p < 0.001 vs. time matched treatment with cytokine alone; two-way ANOVA with Bonferroni corrected multiple comparisons. **B, C** Assessment of apoptosis by Annexin V/PI staining after 48 hours cytokine treatment. **B** Representative dot plots of FITC-fluorescence (x-axis) versus PI-fluorescence (y-axis) are shown. Quadrant representation: Lower left, live cells; lower right, early apoptotic cells; upper left, necrotic cells; upper right, late apoptotic and necrotic cells. **C** Percentage of total apoptotic cells was determined by summation of lower and upper right quadrants and represented as bar graphs. ***p < 0.001 vs. treatment with cytokine alone; one-way ANOVA with Bonferroni corrected multiple comparisons. **D, E** Level of caspase-3 cleavage was assessed by immunofluorescence (IF). **D** Representative IF confocal images after 48 hours cytokine treatment show cleaved caspase-3 positive cells shown in green fluorescence (Alexa488, 40x magnification). **E** Percentages of cleaved caspase-3 positive β cells (cytokines only are 100%) are shown as bar graphs; ***p < 0.001 vs. treatment with cytokine alone; one-way ANOVA with Bonferroni corrected multiple comparisons. Data represents the mean of at least 3 independent experiments ± S.E.M.

**Supplementary Figure 3: rINGAP pre-treatment reduces cytokine-induced apoptosis in RINm5F cells.**

RINm5F cells were pre-treated for 2 hours with 1 nM rINGAP and then exposed to cytokines. **A, B** TUNEL staining followed by immunofluorescence analysis was used for detection of apoptotic cells after 72h. **A** Representative confocal images of RINm5F cells show TUNEL positive cells stained in green fluorescence and red PI nuclear staining. **B** Percentages of TUNEL positive cells relative to cytokines only (100%) are shown by bar graph; **C, D** caspase-3 cleavage was assessed by immunofluorescence. **C** Representative confocal images after 48 hours cytokine treatment show cleaved caspase-3 positive cells shown in green fluorescence (Alexa488, 40x magnification). **D** Percentages of cleaved caspase-3 positive β-cells are shown as bar graphs; ***p < 0.001 vs. treatment with cytokine alone; one-way ANOVA with Bonferroni corrected multiple comparisons. All data represent the mean of at least 3 independent experiments ± S.E.M.

**Supplementary Figure 4: INGAP pre-treatment decreases cytokine-induced Nos2 gene expression and NO production in INS-1 cells.**

INS-1 cells were pre-treated with 1 nM rINGAP or 1.67 μM Ingap-p for 2 hours then exposed to cytokines for **A** 6 hours for assessment of Nos2 gene expression by qRT-PCR and B for 24 and 48 hours for measurement of NO in culture medium by Griess assay. Values were normalized to cytokine alone treatment and expressed as **A** fold change or **B** percentage. Data represents the mean of at least 3 independent experiments ± S.E.M. *P < 0.05, **p < 0.01 ***p < 0.001 vs. treatment with cytokines alone; one-way ANOVA with Bonferroni corrected multiple comparisons.

**Supplementary Figure 5: Characterization of cytokine-induced signaling in RINm5F cells in time-course experiments.**

RINm5F were exposed to the cytokine cocktail, or PBS, for the times indicated and total cell lysates were resolved on 10% stain-free gels for Western blot analysis. Blots were probed with phospho-specific antibodies, followed by stripping and re-probing with non-phospho-antibodies. **A** Assessment of NF-κB signaling pathway. Shown are representative blots for phospho-, non-phospho- antibodies and sections of stain-free blots indicating loading. **B** Probing for activation of JAK2/STAT3, JNK and p38 signaling with phospho- and non-phospho-antibodies.

**Supplementary Figure 6: Effect of pharmacological inhibitors on iNOS expression and p65 phosphorylation**.

Rinm5F cells were pretreated for 30 min with the following inhibitors: **A, E** IKK VII (1 and 10 μM, NF-κB signaling); **B** Jak 1 and AG490 (10 μM and 100 μM, respectively, JAK/STAT signaling); **C** SB202190 (1 μg/ml, p38); and **D** SP600125 (50 μM, JNK), followed by exposure to cytokines for 6 h to assess **A-D** iNOS expression or 1 and 6 h to assess **E** p65 phosphorylation. Shown are representative blots of at least three independent experiments.

**Supplementary Figure 7: Assessment of NF-κB signaling in RINm5F cells by Western blotting for p65 phosphorylation**.

**A, B** RINm5F cells were pretreated with Ingap-p prior to addition of cytokines for 6 h. Phosphorylation of p65 was evaluated by Western blot and quantified by densitometry as a ratio of P-p65/total p65, normalized to total protein. **A** Shown are representative blots of 3 independent experiments, with loading indicated by a band of stain-free gel. **B** Data are expressed as a fold change relative to cytokines only and are means of 3 independent experiments ± S.E.M. **C, D** p65 phosphorylation is co-inhibited by rINGAP and the NF-κB inhibitor IKK VII, as shown in **C** representative blots and **D** quantified by densitometry, as above. Data are expressed as a percentage of cytokines only and are means of 3 independent experiments ± S.E.M.
