# Supplementary figures and images for: Islet neogenesis associated protein (INGAP) protects pancreatic β cells from IL-1β and IFNγ-induced apoptosis
Source: Cell Death Discov. 2021 Mar 17;7:56. doi: 10.1038/s41420-021-00441-z (PMC7969959; doi:10.1038/s41420-021-00441-z)

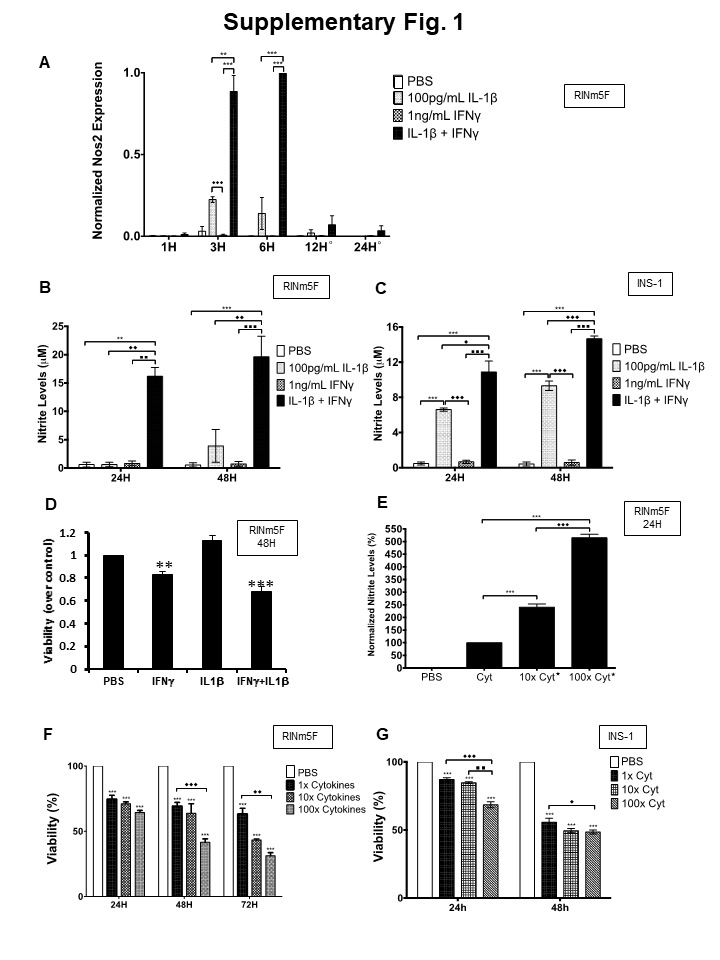

Supplement: Supplementary file 5 — Supplementary Fig. 1 [file 41420_2021_441_MOESM5_ESM.tif]

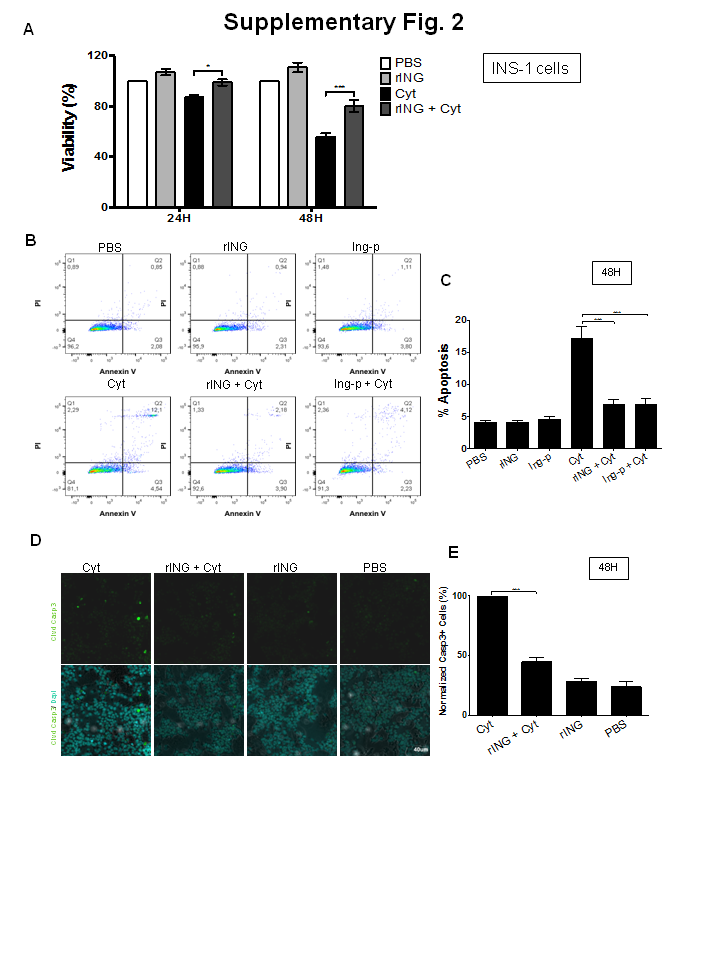

Supplement: Supplementary file 6 — Supplementary Fig. 2 [file 41420_2021_441_MOESM6_ESM.tif]

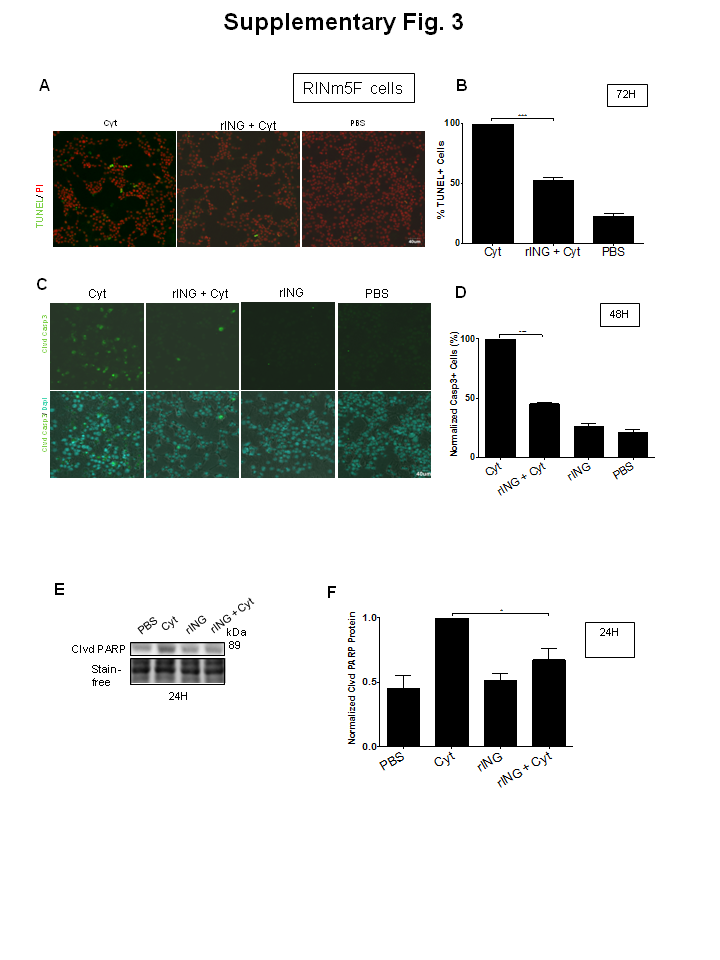

Supplement: Supplementary file 7 — Supplementary Fig. 3 [file 41420_2021_441_MOESM7_ESM.tif]

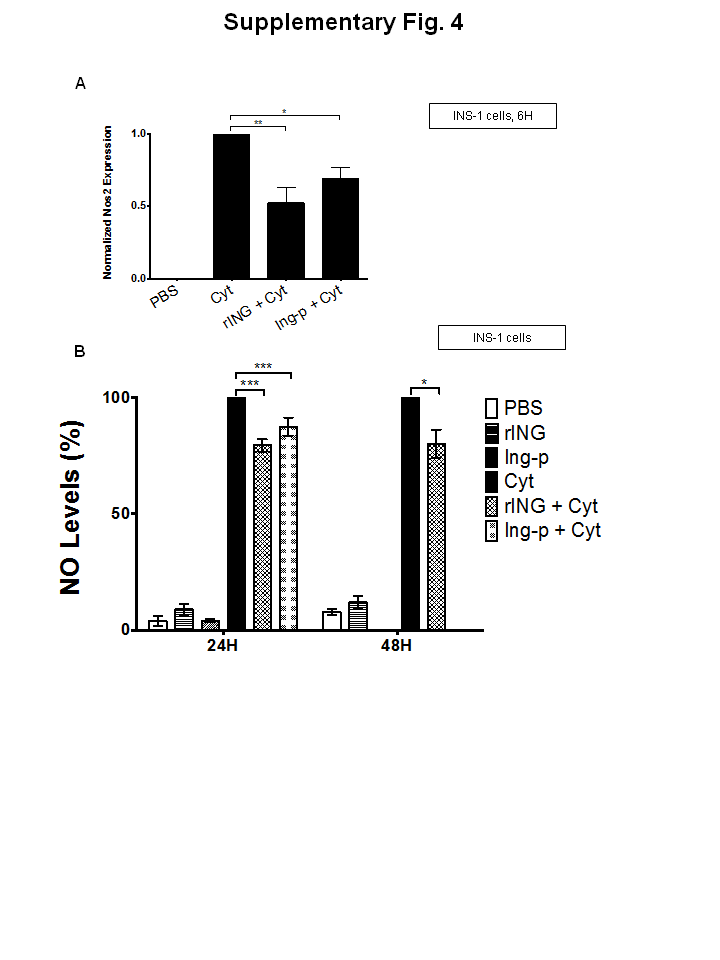

Supplement: Supplementary file 8 — Supplementary Fig. 4 [file 41420_2021_441_MOESM8_ESM.tif]

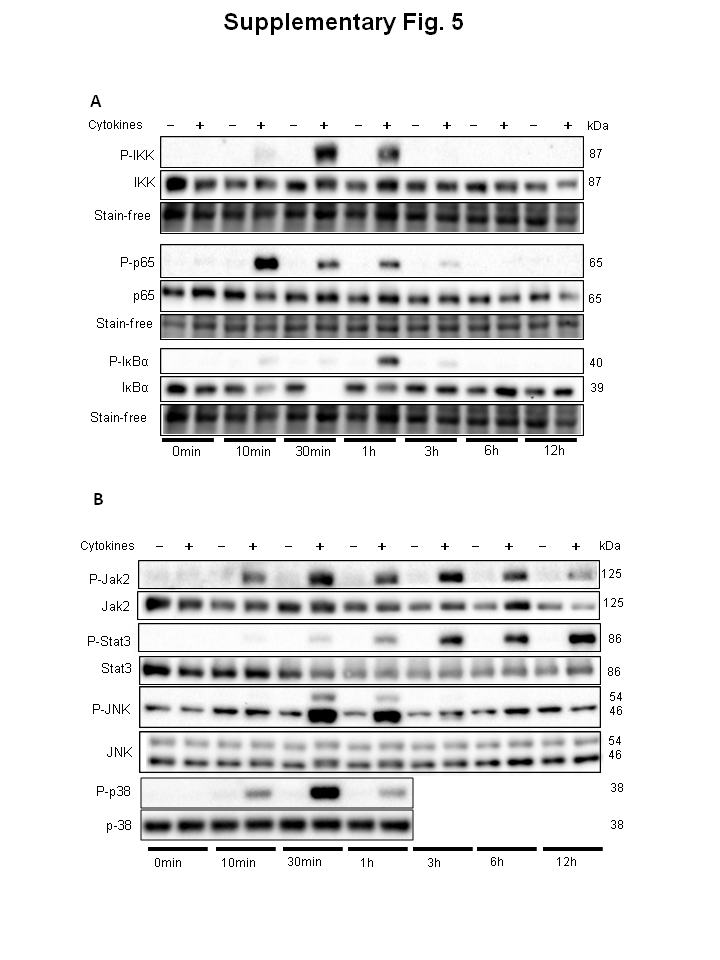

Supplement: Supplementary file 9 — Supplementary Fig. 5 [file 41420_2021_441_MOESM9_ESM.tif]

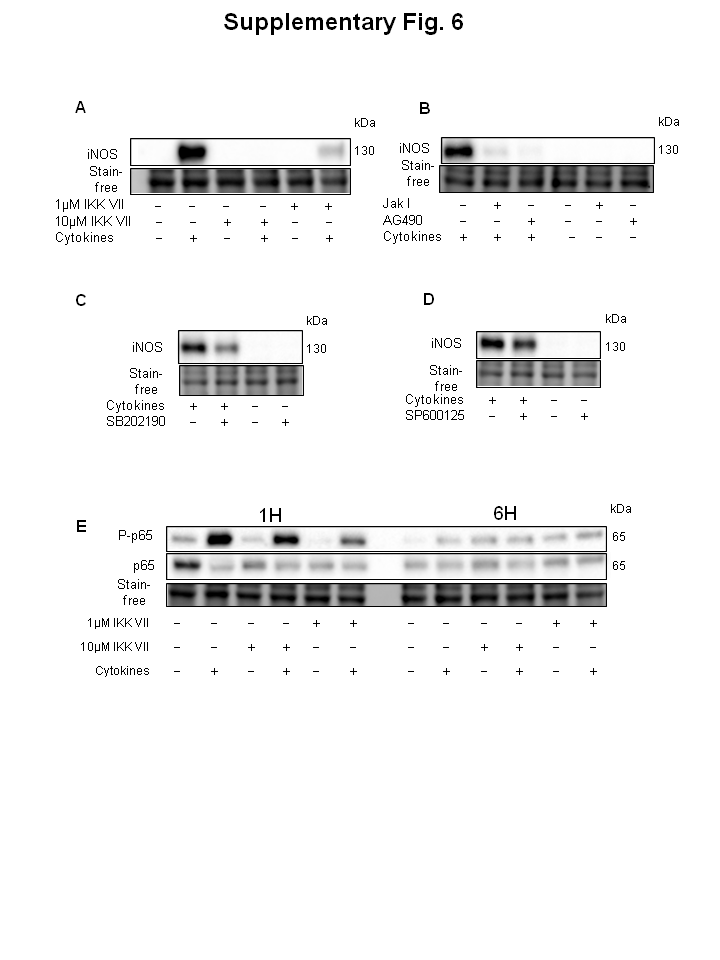

Supplement: Supplementary file 10 — Supplementary Fig. 6 [file 41420_2021_441_MOESM10_ESM.tif]

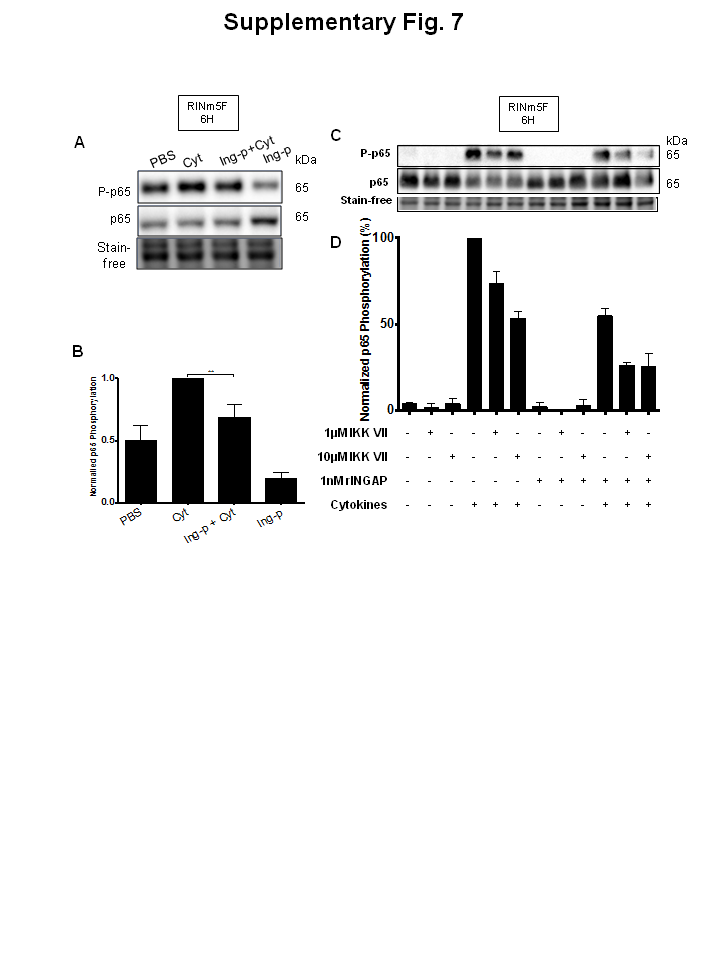

Supplement: Supplementary file 11 — Supplementary Fig. 7 [file 41420_2021_441_MOESM11_ESM.tif]
